# Supplementary material for: Isolation and Identification of Bacteria of Genus Bacillus from Composting Urban Solid Waste and Palm Forest in Northern Peru
Source: Microorganisms. 2023 Mar 15;11(3):751. doi: 10.3390/microorganisms11030751 (PMC10055787; doi:10.3390/microorganisms11030751)
Supplement: Supplementary file 1 [file microorganisms-11-00751-s001.zip › Table S1- Genbank.pdf]

Table S1. List of taxa used in molecular analyses along with strain code followed by accession number, collection locality, date, and source of isolation. GenBank accession numbers under each marker; if marker not sequenced indicated by "–". Sequences generated in present study are in bold.

Species, Collection site, date, source of isolation and strain code	16S	*gyr*A	*rpo*B	Reference
Bacillu cereus				
Tuctilla, Amazonas, Peru; 30-Nov-2020; isolated from compost; Tc-5-68	**OP451872**	–	–	This study
Tuctilla, Amazonas, Peru; 30-Nov-2020; isolated from compost; Tc-2-30	**OP451876**	–	–	This study
Tuctilla, Amazonas, Peru; 30-Nov-2020; isolated from compost; Tc-2-33	**OP451879**	–	–	This study
Tuctilla, Amazonas, Peru; 30-Nov-2020; isolated from compost; Tc-5-66	**OP452926**	–	–	This study
Strain HYS02	MF101471	–	–	Unpublished
Malaysia; 09-Oct-2015; isolated from the host beebread; BD2	KY773595	–	–	Unpublished
solated from soil rhizosphere; strain Neha	MF977311	–	–	Unpublished
Bacillu wiedmannii				
Tuctilla, Amazonas, Peru; 30-Nov-2020; isolated from compost; Tc-2-28	**OP451869**	–	–	This study
Tuctilla, Amazonas, Peru; 30-Nov-2020; isolated from compost; Tc-2-29	**OP451870**	–	–	This study
Tuctilla, Amazonas, Peru; 30-Nov-2020; isolated from compost; Tc-4-47	**OP451871**	–	–	This study
Tuctilla, Amazonas, Peru; 30-Nov-2020; isolated from compost; Tc-6-82	**OP451873**	–	–	This study
Tuctilla, Amazonas, Peru; 30-Nov-2020; isolated from compost; Tc-2-24	**OP451874**	–	–	This study
Tuctilla, Amazonas, Peru; 30-Nov-2020; isolated from compost; Tc-2-25	**OP451875**	–	–	This study
Tuctilla, Amazonas, Peru; 30-Nov-2020; isolated from compost; Tc-2-26	**OP451877**	–	–	This study
Tuctilla, Amazonas, Peru; 30-Nov-2020; isolated from compost; Tc-2-32	**OP451878**	–	–	This study
Tuctilla, Amazonas, Peru; 30-Nov-2020; isolated from compost; Tc-4-46	**OP451880**	–	–	This study
Tuctilla, Amazonas, Peru; 30-Nov-2020; isolated from compost; Tc-6-81	**OP451881**	–	–	This study
Strain ER6	MT124531	–	–	Unpublished
Japan; 30-Sep-2019; isolated from Homo sapiens; G071	LC515603	–	–	Tanaka et al. (2019)
Bacillus safensis* subsp. *safensis				
Tuctilla, Amazonas, Peru; 30-Nov-2020; isolated from compost; Tc-5-67	**OP452925**	–	**OP435795**	

Indonesia; strain PPK6	MZ734317	–	–	Unpublished
Strain P-NA1-2	MT533923	–	–	Unpublished
Strain IMJ7	MT516334	–	–	Unpublished
Strain Bs5	–	–	JX183162	Branquinho et al. (2012)
Strain Bs19	–	–	JX183175	Branquinho et al. (2012)
Seongsan-ri, Jeju Island, South Korea; Nov-2011; isolated from marine sponge; KCTC 12796BP	–	–	CP018197	Unpublished
Bacillus subtilis				
Ocol Palm Forest, Amazonas, Peru; 23-Feb-2021; isolated from soil forest; Oc-A-10	**OP452854**	**OP435785**	**OP435790**	This study
Tuctilla, Amazonas, Peru; 30-Nov-2020; isolated from compost; Tc-1-16	**OP452856**	**OP435787**	**OP435792**	This study
Tuctilla, Amazonas, Peru; 30-Nov-2020; isolated from compost; Tc-5-62	**OP452857**	**OP435788**	**OP435793**	This study
Tuctilla, Amazonas, Peru; 30-Nov-2020; isolated from compost; Tc-4-42	**OP452858**	**OP435789**	**OP435794**	This study
Morocco; Dec-2019; isolated from Cactus rhizosphere; BMI12	MZ712061	–	–	Unpublished
Morocco; Dec-2019; isolated from Cactus rhizosphere; BMF9	MZ712058	–	–	Unpublished
Morocco; Dec-2019; isolated from Cactus rhizosphere; BM1A	MZ712046	–	–	Unpublished
Manipur, India; 19-Mar-2013; isolated from from fermented bamboo shoot; FB6-3	–	CP032089	–	Unpublished
China; 01-May-2015; isolated from marine sediment; BS155	–	CP029052	–	Unpublished
Assam, Sivasagar, India; 10-Jan-2015; isolated from soil; SR1	–	CP021985	–	Unpublished
Kyiv, Ukraine; 1985; isolated from Hay; UCMB5021	–	–	CP051466	Unpublished
Dushanbe, Tajikistan; 1992; isolated from cotton plant surface sterilized stem; UCMB5121	–	–	CP051465	Unpublished
Bacillus velezensis				
Ocol Palm Forest, Amazonas, Peru; 23-Feb-2021; isolated from soil forest; Oc-E-31	**OP452855**	**OP435786**	**OP435791**	This study
China, Ningxia; host: Wolfberry; HSB1	MT626060	–	–	Unpublished
Strain 2563	MT611594	–	–	Unpublished
Harbin, China; 30-10-2019; isolated from *Populus davidiana* x *Populus alba* var. *pyramidalis*; BY6	–	CP051011	CP051011	Zhang et al. (2021)
Beijing, China; 22-Jun-2017; isolated from carnation; LPL061	–	CP042271	–	Unpublished
South Korea; 20-06-2016; isolated from Ganjang (Korean Soy Sauce); SRCM101368	–	–	CP031694	Bang et al. (2020)

References

Bang MS, Jeong HW, Lee YJ, Lee SC, Lee GS, Kim S, Lee HH, Shin JI, Oh CH. (2020) Complete Genome Sequence of Bacillus velezensis Strain DKU_NT_04, Isolated from a Traditional Korean Food Made from Soybeans (Cheonggukjang). Microbiol Resour Announc. 9(24): e00477-20. https://doi.org/10.1128/MRA.00477-20.

Branquinho R., Meirinhos-Soares L., Carriço J.A., Pintado M., Peixe L.V. (2014) Phylogenetic and clonality analysis of Bacillus pumilus isolates uncovered a highly heterogeneous population of different closely related species and clones. FEMS Microbiology Ecology, 90 (3): 689–698. https://doi.org/10.1111/1574-6941.12426

Tanaka M., Onizuka S., Nakayama J. (2020) Germination and cultural isolation of spore-forming bacteria in human feces by using various bile acids. Scientific Reports 10: 15041. https://doi.org/10.1038/s41598-020-71883-1

Zhang P., Diao J., Xie G., Ma L., Wang L. (2021) A Complete Genome Sequence of the Wood Stem Endophyte Bacillus velezensis BY6 Strain Possessing Plant Growth-Promoting and Antifungal Activities. Biomed Res Int. 3904120, doi: https://doi.org/10.1155/2021/3904120
