# Supplementary material for: Isolation and Identification of Bacteria of Genus Bacillus from Composting Urban Solid Waste and Palm Forest in Northern Peru
Source: Microorganisms. 2023 Mar 15;11(3):751. doi: 10.3390/microorganisms11030751 (PMC10055787; doi:10.3390/microorganisms11030751)
Supplement: Supplementary file 1 [file microorganisms-11-00751-s001.zip › Table S2- Morphological and biochemical features of the isolated bacterial strains.pdf]

Table S2. Morphological and biochemical features of the isolated bacterial strains.

N°	Code	Specie	Cell Lenght (µm)	Cell Width (µm)	CAT	IND	CIT	HEM	GEL
1	TC-2-25*	*B. wiedmanii*	2.68 ± 0.63	0.89 ± 0.20	+	-	-	+	-
2	TC-4-68**	*B. cereus*	3.25 ± 0.58	0.92 ± 0.21	+	-	-	+	-
3	Oc-A-10***	*B. subtilis*	2.24 ± 0.48	0.56 ± 0.13	+	-	-	+	+
4	OC-E-31	*B. velezensis*	2.98 ± 0.57	0.53 ± 0.06	+	-	-	+	-
5	TC-4-67	*B. safensis* subsp. *safensis*	1.81 ± 0.19	0.45 ± 0.12	+	-	-	+	-

Catalase – CAT; Índole – IND; Citrate – CIT; Hemolysin -HEM; Gelatin Hydrolysis – GEL;
* Strains TC-2-28, TC-2-29, TC-3-47, TC-5-82, TC-2-24, TC-2-30, TC-2-26, TC-2-32, TC-3-46, TC-5-81 showed similar trends; ** Strains TC-4-66, TC-2-33, showed similar trends; *** Strains TC-3-42, TC-1-16, TC-4-62 showed similar trends.
Note: "+" in the table represents positive production, "-" means negative production.
