# Supplementary material for: Isolation and Identification of Bacteria of Genus Bacillus from Composting Urban Solid Waste and Palm Forest in Northern Peru
Source: Microorganisms. 2023 Mar 15;11(3):751. doi: 10.3390/microorganisms11030751 (PMC10055787; doi:10.3390/microorganisms11030751)
Supplement: Supplementary file 1 [file microorganisms-11-00751-s001.zip › Table S3- Bacterial screening for amylolytic and cellulolytic activities.pdf]

Table S3a. Bacterial screening for amylolytic and cellulolytic activities at 30 °C

Screening amylolytic at 30 °C	Screening cellulolytic at 30 °C
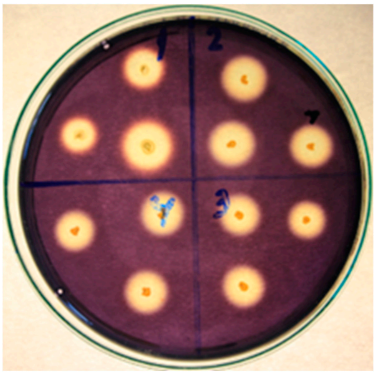	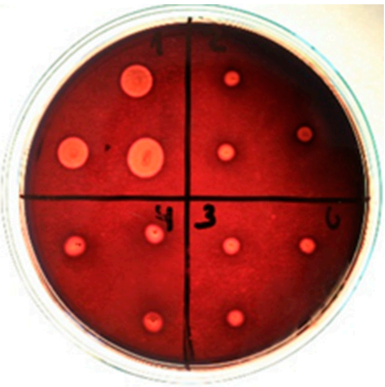
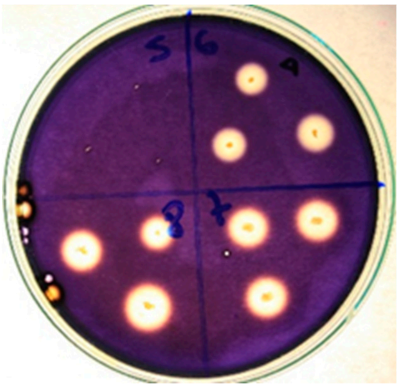	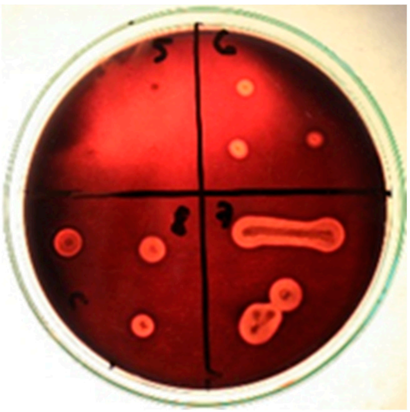

Table S3b. Amylolytic and cellulolytic of isolated strains grow at 15 °C.

Code strain	Amylolytic Index / Halo diameter (mm)	Figure	Cellulolytic Index / Halo diameter (mm)	Figure
TC-2-25	5.33 ± 3.00<br><br>11.29 ± 2.23	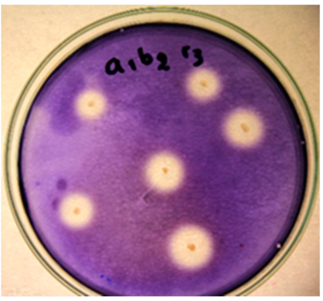	0.74 ± 0.11<br><br>6.53 ± 0.96	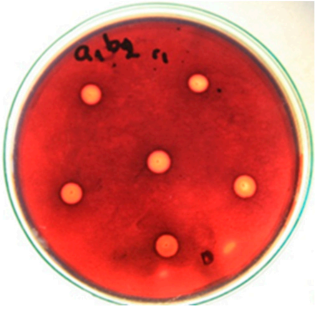
TC-2-28	2.70 ± 0.07<br><br>10.67 ± 1.33	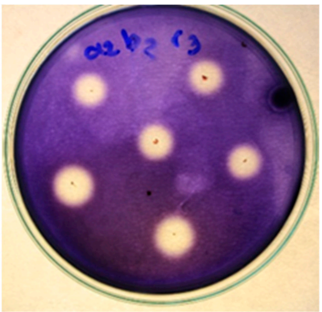	0.79 ± 0.25<br><br>5.66 ± 1.12	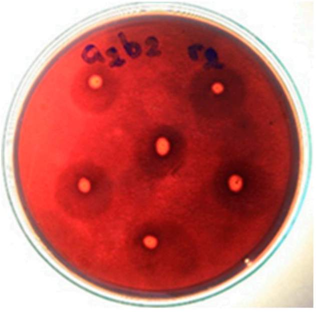
TC-2-29	2.66 ± 0.36<br><br>10.06 ± 1.66	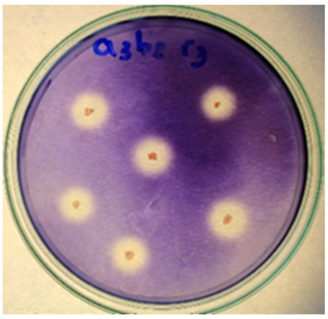	1.08 ± 0.16<br><br>6.33 ± 0.57	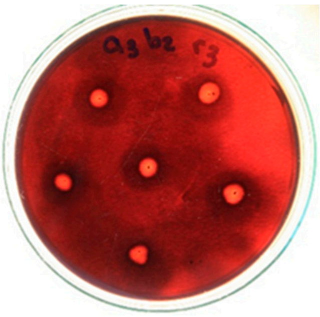
TC-5-82	2.70 ± 0.25<br><br>10.13 ± 1.09	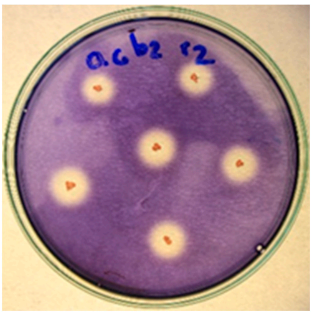	0.46 ± 0.31<br><br>5.34 ± 1.11	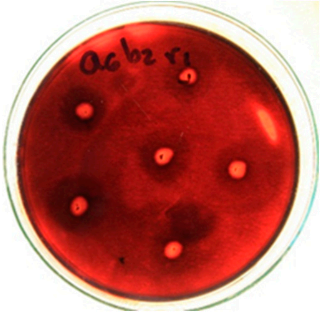

Oc-A-10	2.47 ± 2.77<br>2.7 ± 0.79	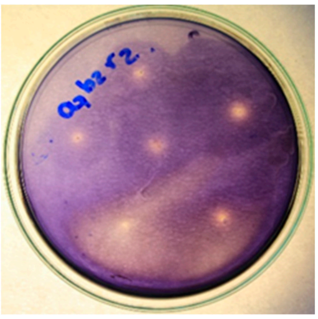	2.15 ± 1.74<br>3.93 ± 0.61	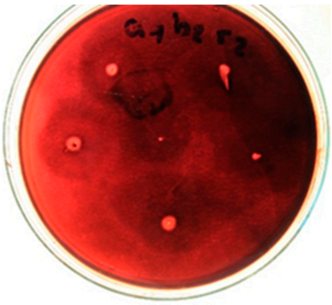
Oc-E-31	0.87 ± 0.10<br>3.09 ± 1.51	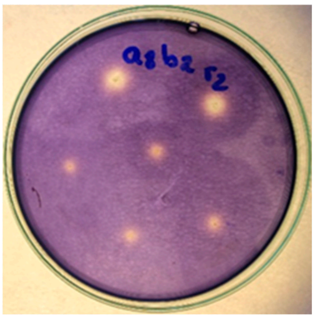	0.97 ± 0.27<br>3.89 ± 0.65	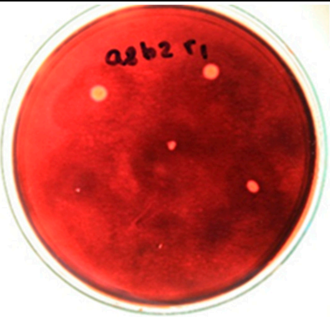
TC-3-42	4.48 ± 0.47<br>6.24 ± 1.39	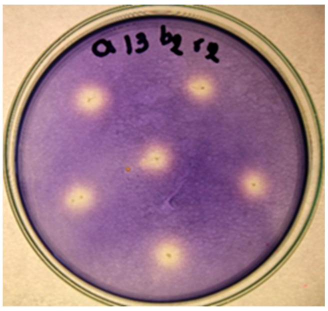	2.52 ± 0.29<br>5.06 ± 0.54	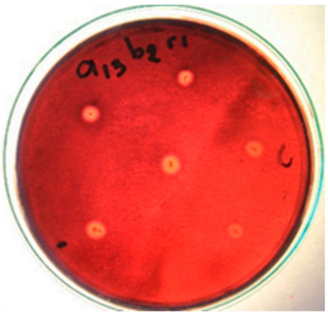

Table S3c. Amylolytic and cellulolytic of isolated strains grow at 20 °C.

Code strain	Amylolytic Index / Halo diameter (mm)	Figure	Cellulolytic Index / Halo diameter (mm)	Figure
TC-2-25	3.16 ± 0.34<br>15.42 ± 1.14	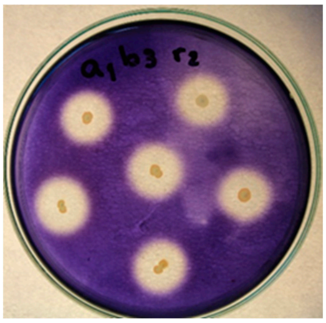	1.15 ± 0.22<br>9.63 ± 1.01	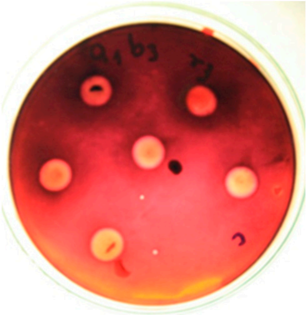
TC-2-28	5.10 ± 0.65<br>16.67 ± 2.20	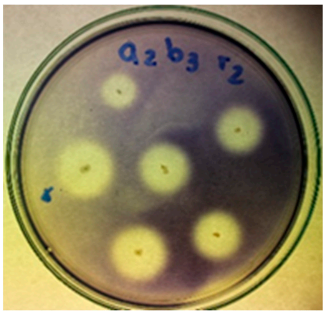	1.12 ± 0.10<br>10.33 ± 1.78	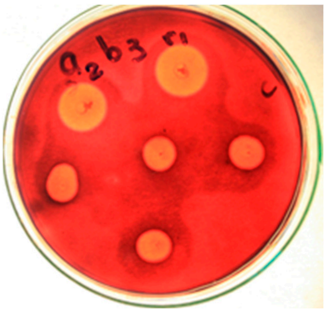
TC-2-29	5.42 ± 0.42<br>17.06 ± 0.87	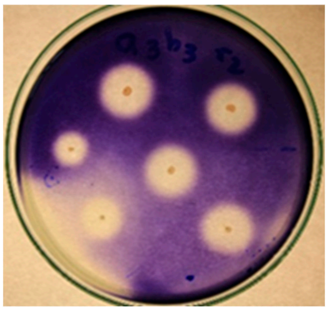	0.90 ± 0.12<br>9.64 ± 0.87	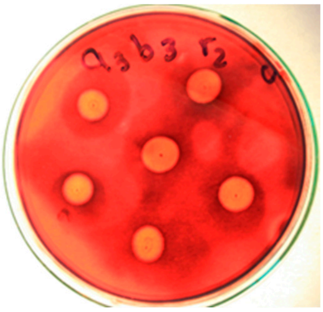
TC-5-82	3.71 ± 0.48<br>16.53 ± 0.70	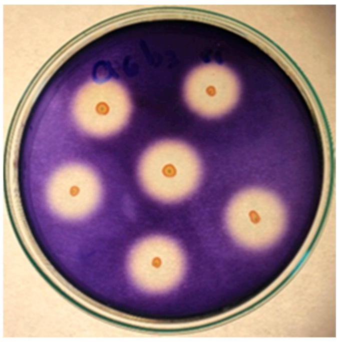	0.97 ± 0.26<br>9.89 ± 0.61	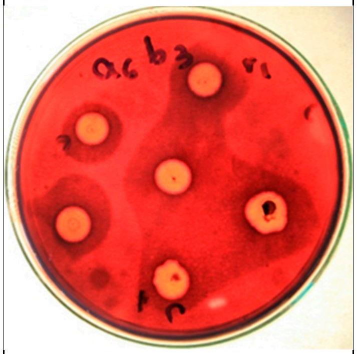

Oc-A-10	2.61 ± 0.30<br><br>11.89 ± 0.90	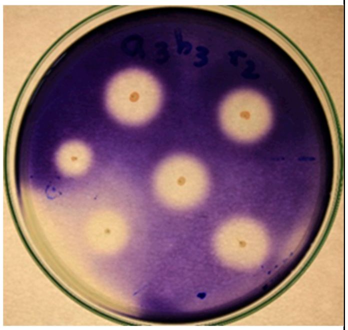	3.04 ± 0.87<br><br>11.25 ± 2.05	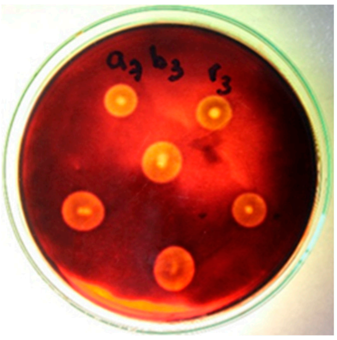
Oc-E-31	4.82 ± 0.34<br><br>18.6 ± 1.83	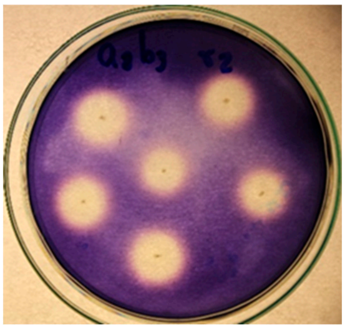	2.96 ± 0.34<br><br>13.25 ± .32	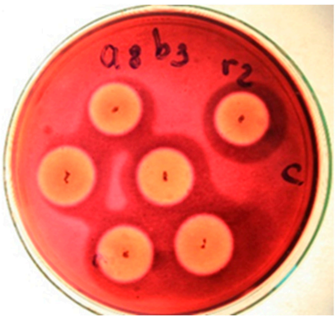
TC-3-42	4.60 ± 0.42<br><br>15.71 ± 1.78	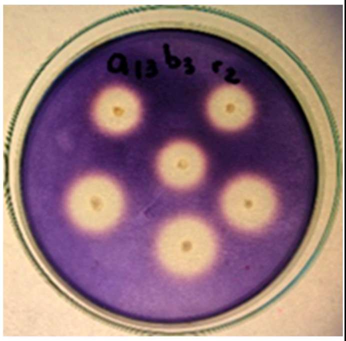	2.77 ± 0.46<br><br>16.28 ± 1.29	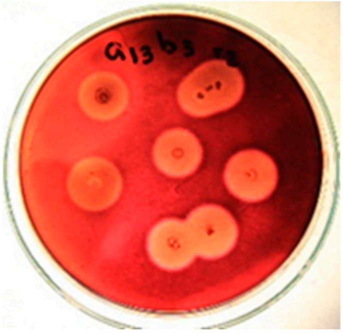

Table S3d. cellulolytic of isolated strains grow at 15 °C.

Code strain	Cellulolytic Index / Halo diameter (mm)	Figure
TC-4-67	3.52 ± 1.72<br><br>5.06 ± 1.22	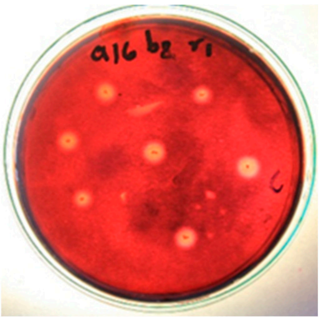
TC-1-16	1.53 ± 1.32<br><br>2.51 ± 0.25	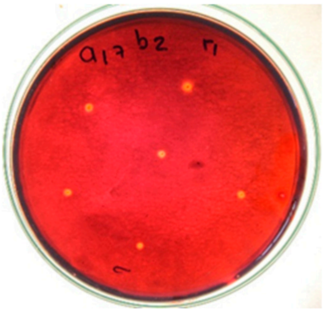

Table S3e. cellulolytic of isolated strains grow at 20 °C.

Code strain	Cellulolytic Index / Halo diameter (mm)	Figure
TC-4-67	5.14 ± 0.81<br><br>13.04 ± 1.29	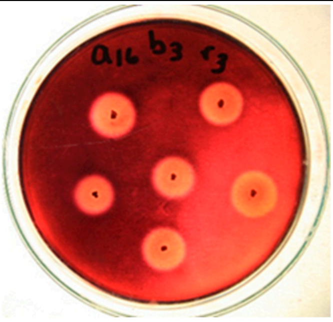
TC-1-16	1.79 ± 0.69<br><br>13.97 ± 2.9	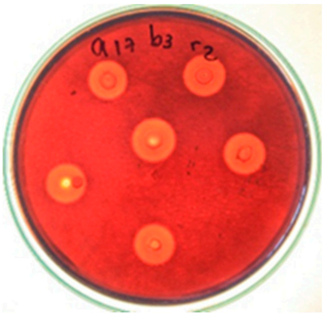
